# Supplementary material for: Urinary Levels of miR-491-5p and miR-592 as Potential Diagnostic Biomarkers in Female Aging Patients with OAB: A Pilot Study
Source: Metabolites. 2022 Aug 31;12(9):820. doi: 10.3390/metabo12090820 (PMC9501158; doi:10.3390/metabo12090820)
Supplement: Supplementary file 1 [file metabolites-12-00820-s001.zip › metabolites-1837997-supplementary.pdf]

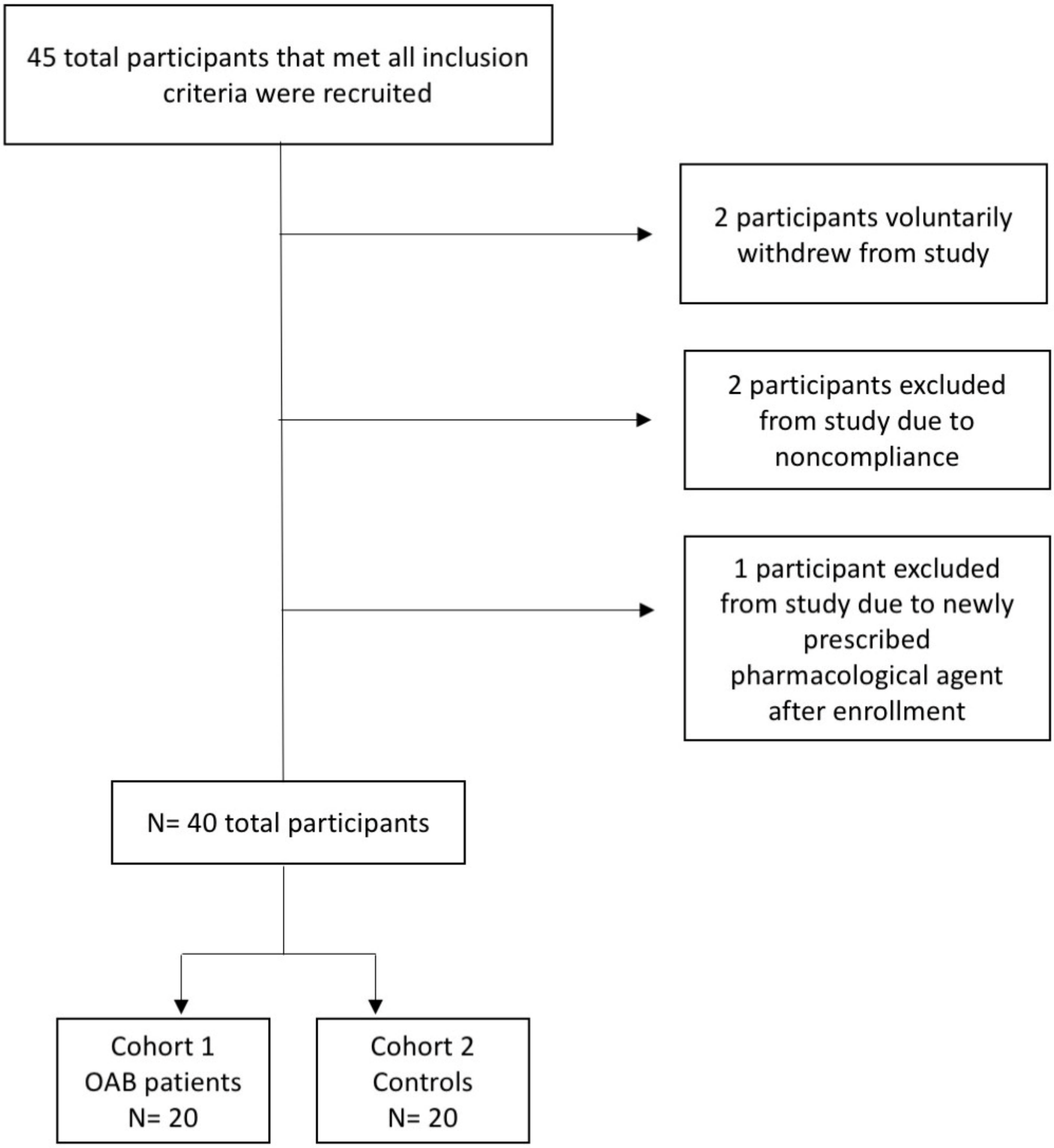

Figure S1. flow diagram.

File S1

Experimental design

The OAB group were female patients who came to urology clinic of the Jewish Hospital for diagnosis and treatment. The control group were female patients attending the clinic without OAB diagnosis or volunteers who agreed to participate in this study as non OAB controls. Each group contains 20 persons. Assays were carried out by the investigator's laboratory, Dr Lysanne Campeau. All authors contributed for 25% of the total work and acknowledge the final version of the paper.

Sample

Samples were first urine of the morning (around 100 mL), in two containers provided by the principal investigator. They were frozen immediately and delivered to the clinic the

following day. Once received, samples were centrifuged, aliquoted and kept at -80C until use.

Nucleic acid extraction

MiRNA were extracted using a specific "Urine microRNA Purification Kit" from Norgen Biotek Corp (Thorold, ON, Canada). Additional reagents not provided in the kit were pure ethanol 100%, provided by the Jewish Hospital Pharmaceutical store and beta-mercap-toethanol (Sigma-Aldrich, Oakville, Canada). No DNAse or RNAse treatment were required. Assessment of contamination by DNA was carried out by using the Nanodrop system for single strand DNA and double strand DNA: no significant amount of DNA was found. RNA contamination was not carried out as the Norgen isolation kit isolates specifically microRNA and small RNAs, the latter being used as a reference to standardize miRNA measurements. Quantification of nucleic acid was carried out on a nanodrop system. Purity of the nucleic acid (RNA) (A260/A280) was close to 2 for all samples.

RNA integrity was assessed by a Nanodrop system.

Reverse transcription

MicroRNAs were first polyadenylated: polyadenylation reactions was prepared as a master mix (Lucigen, Middleton, WI, USA) and adding the following components to a RNase-free 0.2-ml microcentrifuge tubes: 1.0 µl of 10× poly A polymerase buffer (1x recipe below), 1.0 µl of ATP (10 mM), 0.5 µl of E. coli poly(A) polymerase (2 U), 7.5 µl of total extracted RNA. After brief centrifugation, incubation was carried out at 37°C for 30 minutes. Then reaction was terminated at 95°C for 5 minutes, then immediately transferred the tubes to ice.

Subsequently, 5 µl ofRT Oligo-dT Adapter primer containing a polyT sequence (250 nM, sequence 5'- CTCACAGTACGTTGGTATCCTTGTGATGTTCGATGCCATATTGTACTGTGAG-TTTTTT -3') was added and incubated 5 min at 95°C then 5 min at 60°C. After quick spin, the tube was placed on ice for 1+ minutes. Then RT was done using the following Master mix (abm OneSpcriptPlus): 1 µl dNTPs (10 mM), 4.5 µl RT buffer (5x concentrated), 1 µl RT enzyme. 6.5 µl of this mis were added to15 µl of RT template. Then tubes were incubated as follows: RT for 30 minutes, 50°C for 50 minutes and 85°C for 5 minutes. Samples were hold at 4°C, transfer to -20° for long term storage.

qPCR information

	Sequence number
miR-98-5p	MIMAT0000096
let-7b-5p	MIMAT0000063
let-7d-5p	MIMAT0000065
miR-491-5p	MIMAT0002807
miR-885-5p	MIMAT0004947
miR-221-5p	MIMAT0004568
miR-92a-3p	MIMAT0000092
miR-592	MIMAT0003260
miR-21-5p	MIMAT0000076
miR-132	MIMAT0004594
miR-212-5p	MIMAT0022695
snU6.	NR 004394.1

List of primers used for qPCR:

	primers
miR-98-5p	ACACTCCAGCTGGGTGAGGTAGTAAG
let-7b-5p	ACACTCCAGCTGGGTGAGGTAGTAGGT
let-7d-5p	ACACTCCAGCTGGGAGAGGTAGGT
miR-491-5p	ACACTCCAGCTGGGAGTGGGGAACCCT
miR-885-5p	ACACTCCAGCTGGGTCCATTACACTAC
miR-221-5p	ACACTCCAGCTGGGACCTGGCATACAA
92a-3p	ACACTCCAGCTGGGTATTGCACTTGTCCC
miR-592	ACACTCCAGCTGGGTTGTGTCAATATGC
miR-21-5p	ACACTCCAGCTGGGTAGCTTATCAGAC
miR-132	ACACTCCAGCTGGGACCGTGGCTTTCG
miR-212-5p	ACACTCCAGCTGGGACCTTGGCTCTAG
universal primer	CTCACAGTACGTTGGTATCCTTGTG
snU6 forward	CTCGCTTCGGCAGCACATATACT
snU6 reverse	ACGCTTCACGAATTTGCGTGTC

qPCR protocol

Primers were 100 nM (1 µl forward + 1 µl universal primer + 38 µl of water) or 600 nM (6 µl forward + 6 µl universal primer + 28 µl of water) depending on the miRNA studied. The cDNA was 2 µg in 15 µL final, which corresponds to 400 ng per well. The reaction medium per well was 10 µL of Sensifast Mix lo-Rox containing SYBR Green (Fraggabio, Canada), 0.8 µL of primer mix and 6.2 µL of water. In a 96 well plates, 3 µl of sample were added to 17 µL of reaction medium for a total of 20 µL per well. Incubation in the qPCR machine (on an Applied Bioscience 7500 Fast Real-Time PCR) had the following settings: Denature 95°C for 10 minutes, Cycling (45x) 95°C for 15 seconds then 60°C for 35 seconds. Melt curve was carried out to check the presence of a single peak of amplification. Analysis of the data relies on the delta-delta-CT method.

The qPCR validation was carried out using control without cDNA.

Every primer was tested for specificity as follows. The cDNA was prepared at a concentration of 1 µg per 7.5 µl then diluted to 1/0, 1/100 and 1/1000 in water. 3 microL of dilution were used per well in triplicate, to which 17 µL of reaction mix containing the primer of interest was added. Standard curve Ct values = f (cDNA dilution) were plotted. Slopes were computed and close to 3.33 with r2 close to 1 and PCR efficiency close to 2.

Data analysis

The qPCR analysis program was provided with the Applied Bioscience 7500 Fast Real-Time PCR. Cq method determination using the same threshold for all miRNAs examined.

Data were normalized to the amount of snU6 Ct values. Excel datasheet were used for qPCR computation and SPSS for statistical analysis.
